# Prognostic Biomarkers for Predicting Decompensation in Alcoholic and Nonalcoholic Patients With Compensated Cirrhosis: An Umbrella Review

**DOI:** 10.1155/bri/9919068

**Published:** 2026-02-02

**Authors:** Kristina Baktikulova, Saulesh Kurmangaliyeva, Amin Tamadon, Kairat Kurmangaliyev, Nadiar M. Mussin, Ramazon Safarzoda Sharoffidin

**Affiliations:** ^1^ Department of Microbiology, Virology and Immunology, West Kazakhstan Marat Ospanov Medical University, Aktobe, Kazakhstan; ^2^ Department of Natural Sciences, West Kazakhstan Marat Ospanov Medical University, Aktobe, Kazakhstan; ^3^ Department of Surgery No. 2, West Kazakhstan Marat Ospanov Medical University, Aktobe, Kazakhstan; ^4^ State Educational Institution, Avicenna Tajik State Medical University, Dushanbe, Tajikistan, tajmedun.tj

**Keywords:** biomarkers, liver cirrhosis, meta-analysis as topic, prognosis, risk assessment

## Abstract

**Introduction:**

Compensated cirrhosis carries a significant risk of progression to decompensation, which substantially worsens prognosis. Accurate prediction of decompensation events is critical for guiding surveillance, optimizing intervention timing, and improving patient outcomes. Although many prognostic biomarkers have been studied, findings remain heterogeneous. This umbrella review synthesizes evidence from systematic reviews and meta‐analyses to identify and appraise biomarkers predicting decompensation in alcoholic and nonalcoholic compensated cirrhosis.

**Methods:**

PubMed, Scopus, and Web of Science were searched to August 15, 2025, for English‐language systematic reviews and meta‐analyses. From the included meta‐analyses, prognostic performance, heterogeneity, and publication bias were recorded.

**Results:**

Four systematic reviews were included, two with meta‐analyses. Strong predictors across reviews were serum albumin, INR, bilirubin, platelet count, and liver stiffness measurement. HVPG remained a robust invasive predictor, while emerging biomarkers—interleukin‐6, keratin‐18, and extracellular vesicles—were associated with an increased risk of decompensation, although the certainty of evidence was limited by heterogeneity and methodological constraints. Composite scores enhanced predictive accuracy. AMSTAR 2 ratings ranged from high to low, with common reporting limitations. Overlap analysis indicated moderate redundancy among primary studies.

**Conclusions:**

Both established and emerging biomarkers predict decompensation in compensated cirrhosis. Integrated multidomain models combining clinical, biochemical, and imaging‐derived measures may provide the greatest predictive value for guiding clinical decision‐making.

## 1. Introduction

Globally, the burden of cirrhosis and other chronic liver diseases remains substantial; in 2021, prevalence was estimated at approximately 1.70 billion and deaths at about 1.43 million, with an evolving etiologic landscape increasingly influenced by metabolic liver disease [1]. Cirrhosis represents the final common pathway of chronic liver injury and is characterized by progressive fibrosis, architectural distortion, and impaired hepatic function [2]. Although compensated cirrhosis may remain clinically silent for years, the transition to decompensation—manifested by complications, such as ascites, hepatic encephalopathy, variceal bleeding, and, in some cases, hepatocellular carcinoma—marks a dramatic worsening in prognosis, with mortality risk increasing substantially after the first decompensating event [3]. The ability to accurately predict which patients are at highest risk for decompensation is therefore critical for optimizing surveillance, tailoring interventions, and improving clinical outcomes [4].

Prognostic biomarkers, encompassing serum‐based laboratory tests, imaging‐derived measures, hemodynamic parameters, composite scoring systems, and genetic or molecular markers, have been extensively investigated as tools to stratify decompensation risk in compensated cirrhosis [5]. Established predictors, such as the Model for End‐Stage Liver Disease (MELD) score, Child–Pugh classification, liver stiffness measurement (LSM), and hepatic venous pressure gradient (HVPG), are widely used in clinical practice [6]. In recent years, emerging markers—including inflammatory mediators, extracellular vesicles, and disease‐specific genetic variants—have shown promise for refining prognostic models and enabling more individualized risk assessment [7]. Cirrhosis arises from diverse etiologies, including alcohol‐related liver disease (ALD), metabolic dysfunction–associated steatotic liver disease (MASLD), previously referred to as nonalcoholic fatty liver disease (NAFLD) and nonalcoholic steatohepatitis (NASH), chronic viral hepatitis (hepatitis B and C), and less common cholestatic or autoimmune liver diseases [8]. These etiologic pathways differ in their underlying pathophysiology, natural history, and risk of progression, which may influence the performance and applicability of prognostic biomarkers for predicting decompensation [9].

Progression from compensated to decompensated cirrhosis is accompanied by profound metabolic alterations, particularly affecting carbohydrate and amino acid metabolism [10]. Impaired hepatic gluconeogenesis and reduced glycogen storage capacity predispose patients to fasting hypoglycemia, while disrupted amino acid metabolism contributes to sarcopenia, altered branched chain‐to‐aromatic amino acid ratios, and impaired nitrogen handling [11]. These metabolic disturbances are closely linked to hyperammonemia, systemic inflammation, and neurotoxicity, which play key roles in the development of complications, such as ascites and hepatic encephalopathy [12]. Recognition of these metabolic changes provides a mechanistic rationale for evaluating biochemical, inflammatory, and composite biomarkers as predictors of decompensation risk [13].

However, the evidence base for these biomarkers is heterogeneous, reflecting variability in study design, patient populations, etiology‐specific pathophysiology, outcome definitions, and statistical methods [14]. Multiple systematic reviews and meta‐analyses have synthesized findings in this area, but their scope, methodological rigor, and conclusions differ. This variability can make it challenging for clinicians and policymakers to discern which biomarkers are most reliable and applicable in different settings.

Umbrella reviews, which systematically collate and critically appraise evidence from multiple systematic reviews and meta‐analyses, offer a high‐level synthesis that can clarify the strength and consistency of the available data [15]. To our knowledge, no prior umbrella review has comprehensively examined prognostic biomarkers for predicting decompensation in compensated cirrhosis across both alcoholic and nonalcoholic etiologies.

Beyond summarizing existing systematic reviews, this umbrella review provides a higher order synthesis by evaluating cross‐review consistency, grading the overall strength of evidence for each biomarker, and integrating methodological quality and overlap of primary studies. This approach allows clinicians to distinguish biomarkers supported by robust, reproducible evidence from those with promising but uncertain prognostic value. By translating heterogeneous findings into an evidence strength–based framework, this review aims to support more informed clinical risk stratification in compensated cirrhosis.

Therefore, the objective of this umbrella review was to identify, summarize, and appraise the highest level evidence on prognostic biomarkers and prediction models for decompensation in compensated cirrhosis, with a focus on etiology‐specific performance, methodological quality, and implications for clinical practice and future research.

Although the number of eligible systematic reviews is limited, an umbrella review enables a higher order synthesis by comparing the consistency of findings across reviews, evaluating overlap of primary studies, and grading the overall strength of evidence for each biomarker. This approach allows differentiation between biomarkers supported by consistent, methodologically robust evidence and those with promising but uncertain prognostic value. Such stratification is not achievable by individual reviews alone.

## 2. Methods

### 2.1. Guideline and Protocol

This umbrella review was conducted in accordance with the Preferred Reporting Items for Systematic Reviews and Meta‐Analyses (PRISMA) 2020 statement, ensuring transparency, completeness, and reproducibility of the review process. The methodology followed the recommendations for conducting umbrella reviews as described by the Joanna Briggs Institute (JBI) and adhered to the guidance for synthesizing evidence from systematic reviews and meta‐analyses.

The protocol specified that only systematic reviews and meta‐analyses evaluating prognostic biomarkers or prediction models for the risk of decompensation in adult patients with compensated cirrhosis—of alcoholic or nonalcoholic etiology—would be included. It also outlined the use of validated tools for assessing the methodological quality of the included reviews (AMSTAR 2) and the primary studies therein, as well as the application of the GRADE approach to assess the certainty of evidence.

This umbrella review synthesized evidence from previously published systematic reviews and meta‐analyses and did not involve the collection of individual participant data. Therefore, institutional ethical approval and informed consent were not required.

### 2.2. Inclusion and Exclusion Criteria

#### 2.2.1. Search Strategy

A comprehensive literature search was conducted across PubMed/MEDLINE, Scopus, and Web of Science up to August 15, 2025, with language restrictions on English articles, to identify systematic reviews and meta‐analyses on prognostic biomarkers for predicting decompensation in alcoholic and nonalcoholic patients with compensated cirrhosis. Boolean operators were used to refine the search strategy. Additional backward and forward citation tracking of included articles was performed to minimize selection bias. The full search strategy is available in Table 1 to ensure methodological transparency and reproducibility.

**TABLE 1:** Search strategy for the umbrella review of prognostic biomarkers predicting decompensation in alcoholic and nonalcoholic compensated cirrhosis across PubMed, Scopus, and Web of Science.

Code	Search query
#1	TITLE‐ABS‐KEY (“compensated cirrhosis” OR “compensated liver cirrhosis”)
#2	TITLE‐ABS‐KEY (“biomarker” OR “prognostic marker” OR “serum marker”)
#3	TITLE‐ABS‐KEY (“decompensation” OR “ascites” OR “hepatic encephalopathy” OR “variceal bleeding”)
#4	TITLE‐ABS‐KEY (“hazard ratio” OR “risk factor” OR “prognosis”)
#5	TITLE‐ABS‐KEY (“systematic review” OR “meta‐analysis”)
#6	#1 AND #2 AND #3 AND #4 AND #5

#### 2.2.2. Literature Screening and Data Extraction

All records retrieved from the database searches were imported into EndNote X9 (Clarivate Analytics) for reference management, and duplicates were identified and removed. The screening process was performed in two sequential stages using Rayyan QCRI (Qatar Computing Research Institute) to facilitate blinded review and reduce selection bias.

In the first stage (title and abstract screening), two reviewers (K.B. and S.K.) independently assessed all retrieved citations against the predefined inclusion and exclusion criteria. Articles that clearly did not meet eligibility requirements were excluded at this stage.

In the second stage (full‐text screening), the same reviewers independently evaluated the full texts of potentially eligible studies. Disagreements at either stage were resolved through discussion, and when consensus could not be reached, a third reviewer (A.T.) acted as an adjudicator. The reasons for full‐text exclusions were documented to ensure transparency.

For all included systematic reviews and meta‐analyses, data were extracted independently by two reviewers (K.B. and K.K.) using a piloted standardized extraction form in Microsoft Excel.

Where meta‐analyses were available, extracted data included hazard ratios (HR), odds ratios (OR), area under the receiver operating characteristic curve (AUROC), or other relevant prognostic performance measures, along with their 95% confidence intervals (CIs) and heterogeneity statistics (*I* ^2^ and *τ* ^2^).

When essential data were missing or unclear, attempts were made to retrieve additional information from supporting files or by contacting corresponding authors. All extracted data were cross‐checked for accuracy and consistency before being tabulated for synthesis.

#### 2.2.3. Quality Assessment

The methodological quality of each included systematic review and/or meta‐analysis was independently evaluated by two reviewers (S.K. and K.K.) using the A MeaSurement Tool to Assess systematic Reviews, Version 2 (AMSTAR 2). This tool assesses critical domains, such as protocol registration, comprehensiveness of the literature search, justification for excluding studies, risk‐of‐bias assessment for included studies, appropriateness of meta‐analytical methods, and consideration of publication bias. Reviews were categorized as having high, moderate, low, or critically low methodological quality based on AMSTAR 2’s overall rating guidelines.

For systematic reviews that incorporated prediction models, the Prediction model Risk Of Bias ASsessment Tool (PROBAST) was applied to evaluate risk of bias and applicability across four domains: participants, predictors, outcomes, and analysis. For systematic reviews of prognostic biomarkers without prediction model development, the Quality in Prognosis Studies (QUIPS) tool was used to assess the risk of bias of the primary studies included in those reviews.

Each domain was rated as low, moderate, or high risk of bias according to the scoring criteria of the respective tool. Disagreements between reviewers were resolved by discussion, and if needed, adjudicated by a third reviewer (A.T.).

Where available, the certainty of evidence for each prognostic biomarker or model was extracted directly from the included reviews if reported using the Grading of Recommendations Assessment, Development, and Evaluation (GRADE) approach. If not reported, certainty was appraised by the review team based on GRADE principles, considering factors, such as study limitations, consistency of results, directness of evidence, precision, and publication bias.

#### 2.2.4. Statistical Analysis

Given that this study is an umbrella review, statistical synthesis relied primarily on the pooled estimates and analyses reported in the included meta‐analyses. No new meta‐analysis of primary studies was performed. When multiple systematic reviews reported meta‐analytic results for the same biomarker or prediction model, the effect sizes (e.g., HR, OR, and AUROC/C‐statistic) and their corresponding 95% CIs were extracted directly from the original meta‐analyses.

Where effect sizes were available, the type of statistical model used (random‐effects or fixed‐effects), heterogeneity measures (*I* ^2^ and *τ* ^2^), and publication bias assessments (e.g., Egger’s test and funnel plots) were recorded. For meta‐analyses using HR, results were presented on the log scale when necessary for consistency of reporting.

In cases where multiple meta‐analyses assessed the same biomarker, narrative synthesis was used to compare effect sizes, heterogeneity, and the robustness of results across reviews. Overlap analysis was conducted using the corrected covered area (CCA) method to quantify the degree of primary study duplication between included reviews.

Where narrative synthesis was required (e.g., for systematic reviews without meta‐analysis), results were organized by biomarker category (serum‐based, imaging‐based, composite, genetic, inflammatory, hemodynamic, etc.) and by etiology (alcoholic vs. nonalcoholic), with emphasis on consistency, strength of association, and predictive performance.

All extracted statistical results were tabulated, and summary of findings tables were created to present prognostic performance measures alongside quality assessment outcomes (AMSTAR 2, QUIPS/PROBAST, and GRADE). Statistical data reported in the included reviews were not recalculated or repooled, in accordance with best practices for umbrella reviews.

#### 2.2.5. Evidence Strength Classification Framework

An a priori framework was applied to classify the strength of evidence for each prognostic biomarker synthesized in this umbrella review. Strong evidence was defined as a biomarker demonstrating directionally consistent associations with decompensation across at least two systematic reviews and/or meta‐analyses, supported by at least moderate certainty of evidence (GRADE ≥ moderate), without prohibitive heterogeneity (*I* ^2^ ≤ 75% or heterogeneity explained by predefined subgroup or etiology analyses), and without a predominance of high risk of bias in primary studies or prediction models (QUIPS/PROBAST).

Moderate evidence was assigned when associations were directionally consistent but limited by low certainty of evidence (GRADE low), substantial unexplained heterogeneity (*I* ^2^ > 75%), or notable concerns regarding risk of bias. Weak or insufficient evidence was defined as evidence derived from a single review, inconsistent direction of effect, very high unexplained heterogeneity, serious methodological limitations, or sparse outcome data. This framework was applied uniformly across biomarker categories to support transparent interpretation and clinical relevance.

## 3. Results

### 3.1. Characteristics of Included Studies

**Figure 1:**
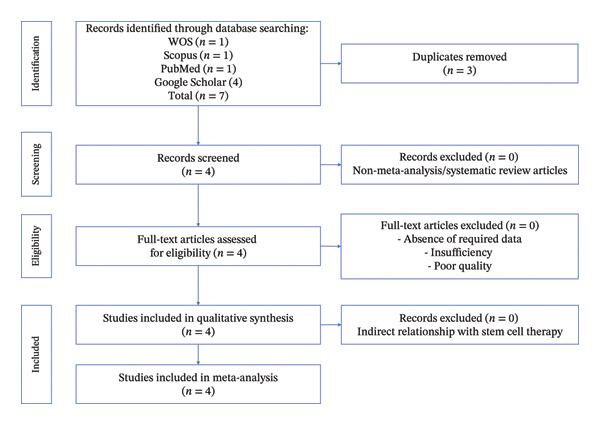
PRISMA 2020 flow diagram illustrating the selection process of systematic reviews and meta‐analyses evaluated prognostic biomarkers for predicting decompensation in alcoholic and nonalcoholic patients with compensated cirrhosis.

This umbrella review included four systematic reviews published between 2024 and 2025 (Figure 1), two of which incorporated meta‐analyses and one that provided a narrative synthesis only (Table 2, S1 and S2). These reviews were published in both hepatology‐focused and broader medical journals and evaluated prognostic biomarkers or prediction models for decompensation in adult patients with compensated cirrhosis. The number of primary studies included in the reviews ranged from 16 to 66, with total participant numbers varying from unreported figures to as many as 37,063 individuals. Study populations were typically drawn from observational cohorts, case–control studies, and control arms of randomized controlled trials, and most were defined using the Baveno VII criteria or equivalent definitions for compensated cirrhosis.

**Table 2:** Overview of included systematic reviews and meta‐analyses on prognostic biomarkers for predicting decompensation in compensated cirrhosis.

Author, year (ref.)	Journal	Review type	Population	Etiology focus	Decompensation outcome (s)	Main conclusion
Amoroso et al., 2024 [16]	Journal of Molecular Medicine	SR	Adults with NAFLD/NASH cirrhosis (F3–F4)	NAFLD–NASH	Composite liver‐related events	Several noninvasive biomarkers (e.g., LSM, ELF, and APRI) are associated with decompensation risk; ethnicity‐specific thresholds may be required.
Baktikulova et al., 2025 [9]	Frontiers in Medicine	SR + MA	Adults with compensated cirrhosis (Baveno VII)	ALD and NAFLD–NASH	Ascites, HE, and VB	Etiology‐specific patterns observed: Structural/functional markers performed better in NAFLD–NASH, while inflammatory markers showed stronger associations in ALD.
Gananandan et al., 2024 [17]	BMJ Open Gastroenterology	SR + MA	Adults with compensated cirrhosis	Mixed etiologies	Ascites, HE, and VB	Albumin and INR were the most consistent laboratory markers; imaging and HVPG improved risk stratification.
Haghnejad et al., 2025 [18]	Hepatology	SR	Adults with compensated advanced chronic liver disease	Mixed etiologies	First decompensation (various definitions)	Several prediction models showed moderate discrimination, but most were at high risk of bias and lacked external validation; none were ready for routine clinical use.

*Note:* Detailed biomarker lists, pooled estimates, heterogeneity metrics, and methodological assessments are provided in Supporting Table S1. ALD, alcohol‐related liver disease; ELF, enhanced liver fibrosis score; NASH, nonalcoholic steatohepatitis.

Abbreviations: HE, hepatic encephalopathy; HVPG, hepatic venous pressure gradient; INR, international normalized ratio; LSM, liver stiffness measurement; MA, meta‐analysis; NAFLD, nonalcoholic fatty liver disease; SR, systematic review; VB, variceal bleeding.

The reviews differed in their etiology focus. Amoroso et al. [16] concentrated specifically on NAFLD and NASH‐related cirrhosis, whereas Baktikulova et al. [9] stratified their findings by ALD and NAFLD–NASH. Gananandan et al. [17] included a mixed‐etiology population. Haghnejad et al. [18] likewise examined mixed‐etiology cohorts of compensated advanced chronic liver disease, but noted the absence of models developed specifically for ALD and substantial variability in outcome definitions across studies. Across the reviews, decompensation outcomes most commonly assessed included ascites, hepatic encephalopathy, and variceal bleeding, with some also incorporating hepatocellular carcinoma or composite liver‐related events.

The spectrum of prognostic biomarkers assessed was broad, spanning serum‐based measures, such as albumin, bilirubin, platelets, international normalized ratio (INR), neutrophil‐to‐lymphocyte ratio, keratin‐18, and alpha‐fetoprotein; imaging‐based parameters including LSM, magnetic resonance elastography, spleen size, and liver–spleen ratio; composite scoring systems, such as MELD, Child–Pugh, ALBI, NAFLD fibrosis score, FIB‐4, APRI, and MEFIB; hemodynamic indicators, such as HVPG; inflammatory markers, such as interleukin‐6 and extracellular vesicles; genetic variants, such as the PNPLA3 GG genotype; and endoscopic indicators of portal hypertension. These biomarkers were measured using a combination of serum assays, advanced imaging modalities including transient elastography, MRI, CT, and Doppler ultrasound, hemodynamic measurements, and genetic testing.

The main conclusions of the reviews varied according to their focus. Amoroso et al. [16] identified several noninvasive biomarkers with prognostic value in NASH cirrhosis, particularly ELF, LSM, APRI, and MEFIB, and noted the importance of ethnicity‐specific thresholds. Baktikulova et al. [9] reported that structural and functional markers performed best in NAFLD–NASH, while inflammatory markers, such as extracellular vesicles and keratin‐18, were stronger predictors in ALD; interleukin‐6 was predictive in both etiologies. Gananandan et al. [17] found that INR and albumin were the strongest serum predictors of decompensation, with imaging measures and HVPG showing high reliability and novel composite scores offering additional promise. Overall, the included reviews provided complementary evidence on the utility of diverse prognostic biomarkers for anticipating decompensation events in patients with compensated cirrhosis. Haghnejad et al. [18] systematically appraised multivariable prediction models for first decompensation in compensated advanced chronic liver disease across diverse etiologies. They found that while several models—particularly those incorporating elastography (e.g., MRE‐based models and ABC algorithm) and composite indices (e.g., ALBI‐FIB4 and ANTICIPATE‐NASH‐LRE)—showed good discriminatory ability (AUC 0.75–0.92), most were limited by methodological weaknesses, heterogeneity in outcome definitions, and reliance on nonroutine predictors. Thirteen of sixteen studies were rated at high risk of bias, and none of the models were deemed ready for clinical implementation. The authors emphasized the need for standardized outcome definitions, routine clinical predictors, and robust external validation before these models can be applied in practice.

Using the predefined evidence strength framework, established laboratory markers (albumin, INR, bilirubin, and platelet count) and LSM were classified as having strong evidence for predicting first decompensation. Hemodynamic assessment by HVPG also met criteria for strong evidence, albeit as an invasive modality. Inflammatory biomarkers (e.g., interleukin‐6 and keratin‐18), extracellular vesicles, and genetic variants, such as PNPLA3, were classified as having moderate or weak evidence, owing to heterogeneity, lower certainty of evidence, and methodological limitations. Table 3 presents a clinician‐facing ranking of prognostic biomarkers based on predefined evidence strength, invasiveness, availability, and etiology‐specific performance.

**Table 3:** Evidence strength–based ranking of prognostic biomarkers for predicting decompensation in compensated cirrhosis.

Biomarker/predictor	Evidence strength	Domain	Invasiveness	Clinical availability	Etiology specificity	Key limitations
Serum albumin	Strong	Laboratory	Noninvasive	Routine	Consistent across ALD and non‐ALD	Influenced by nutrition, inflammation, and fluid status
INR	Strong	Laboratory	Noninvasive	Routine	Consistent across etiologies	Affected by anticoagulants and vitamin K status
Bilirubin	Strong	Laboratory	Noninvasive	Routine	Consistent across etiologies	Less specific in cholestatic disease
Platelet count	Strong	Laboratory	Noninvasive	Routine	Consistent across etiologies	Indirect marker of portal hypertension; nonspecific
Liver stiffness measurement (LSM)	Strong	Imaging	Noninvasive	Widely available (specialized centers)	Strong in MASLD/NAFLD and mixed etiologies	Cut‐off variability; influenced by inflammation and congestion
Hepatic venous pressure gradient (HVPG)	Strong	Hemodynamic	Invasive	Limited to tertiary centers	Consistent across etiologies	Invasive, costly, and limited accessibility
MELD score	Strong	Composite (laboratory‐based)	Noninvasive	Routine	Consistent across etiologies	Not designed specifically for compensated disease
ALBI score	Strong	Composite (laboratory‐based)	Noninvasive	Routine	Strong in NAFLD/MASLD and mixed etiologies	Limited discrimination for specific decompensation types
FIB‐4/APRI	Moderate	Composite (laboratory‐based)	Noninvasive	Routine	Better in viral and metabolic disease	Reduced accuracy in older patients
Spleen size/portal vein diameter	Moderate	Imaging	Noninvasive	Widely available	Mixed etiologies	Operator‐dependent; indirect surrogate
Interleukin‐6 (IL‐6)	Moderate	Inflammatory	Minimally invasive	Limited (research‐oriented)	Predictive in both ALD and non‐ALD	High heterogeneity; assay variability
Keratin‐18 (K18)	Moderate	Cell injury marker	Minimally invasive	Limited	Stronger in ALD	Nonstandardized cut‐offs
Extracellular vesicles (EVs)	Weak–Moderate	Inflammatory/molecular	Minimally invasive	Research only	Stronger signals in ALD	Lack of standardized assays; poor reproducibility
PNPLA3 GG genotype	Weak–Moderate	Genetic	Noninvasive	Limited	NAFLD/NASH‐specific	Not predictive alone; ethnicity‐dependent
Advanced prediction models (ML‐based)	Weak	Multidomain	Noninvasive	Research only	Variable	High risk of bias; limited external validation
Metabolomic/omics signatures	Weak	Molecular	Minimally invasive	Research only	Etiology‐specific	Cost, limited validation, and lack of cut‐offs

*Note:* ALBI, albumin–bilirubin score; ALD, alcohol‐related liver disease; APRI, aspartate aminotransferase to platelet ratio index; EVs, extracellular vesicles; FIB‐4, fibrosis‐4 index; IL‐6, interleukin‐6; K18, keratin‐18 (cytokeratin‐18); MASLD, metabolic dysfunction–associated steatotic liver disease; MELD, Model for End‐Stage Liver Disease; NASH, nonalcoholic steatohepatitis; PNPLA3, patatin‐like phospholipase domain‐containing protein 3.

Abbreviations: HVPG, hepatic venous pressure gradient; INR, international normalized ratio; LSM, liver stiffness measurement; ML, machine learning; NAFLD, nonalcoholic fatty liver disease.

Across the included reviews, most analyses evaluated composite outcomes of first decompensation, while fewer reported event‐specific performance for ascites, variceal bleeding, or hepatic encephalopathy separately. Consequently, direct comparison of biomarker performance by individual decompensation type was limited and inconsistently reported.

### 3.2. Results of Methodological Quality Assessment

The methodological quality of the four included systematic reviews was appraised using the AMSTAR‐2 tool across 16 items, seven of which are considered critical domains (items 2, 4, 7, 9, 11, 13, and 15). A traffic‐light summary of individual item ratings for each review is presented in Figure 2(a), and the distribution of “Yes,” “Unclear,” and “No” responses across AMSTAR‐2 items is shown in Figure 2(b).

Figure 2(a) Traffic‐light heatmap of AMSTAR‐2 assessments across four systematic reviews. Responses for each of the 16 AMSTAR‐2 items are shown for Amoroso et al. [16], Baktikulova et al. [9], Gananandan et al. [17], and Haghnejad et al. [18], and critical domains (items 2, 4, 7, 9, 11, 13, and 15) are listed at the top. (b) Summary of AMSTAR‐2 item ratings by review. Counts and percentages of “Yes,” “Unclear,” and “No” responses across all 16 AMSTAR‐2 items are shown for each review.

**Figure:**
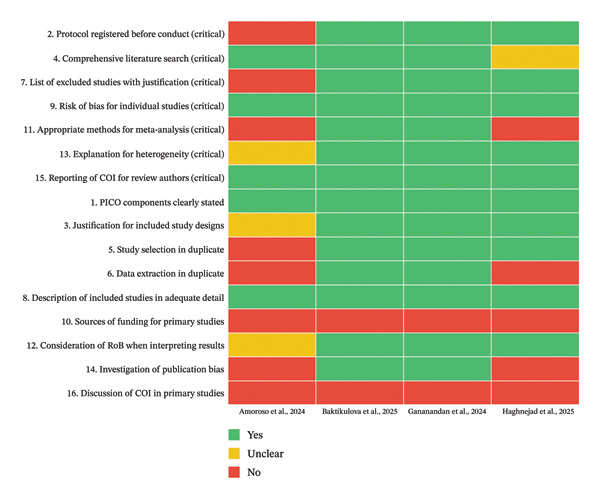
(a)

**Figure:**
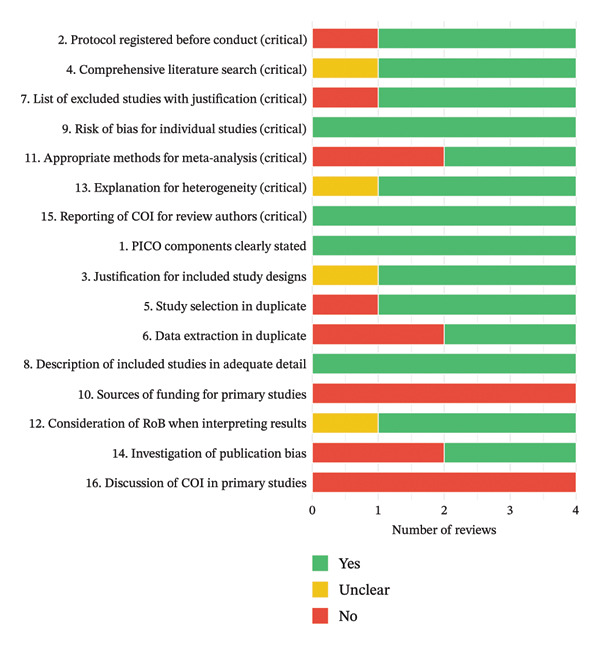
(b)

Overall, Baktikulova et al. [9] met all seven critical domains and most noncritical items, achieving a high confidence rating. The review had a preregistered protocol in PROSPERO, employed duplicate screening and data extraction, provided a list of excluded studies with justifications, used appropriate meta‐analytical methods, investigated publication bias, and considered risk of bias in interpreting results. Minor shortcomings were the absence of reporting on funding sources and conflicts of interest for the primary studies.

Gananandan et al. [17] also demonstrated strong adherence to AMSTAR‐2 standards, with a PROSPERO‐registered protocol, comprehensive search, duplicate screening and extraction, and provision of an excluded studies list. Appropriate statistical synthesis and publication bias assessments were conducted, and risk of bias was incorporated into interpretation. However, approximately one‐quarter of the primary studies were rated at high risk of bias in their QUIPS assessment, and the review did not report funding or conflicts of interest for included primary studies. The overall confidence rating was moderate.

In contrast, Amoroso et al. [16] satisfied fewer critical domains and were rated as low confidence. Although the review addressed the PICO question clearly, performed a comprehensive search, and assessed the risk of bias of included studies, it did not register a protocol, conduct duplicate study selection or data extraction, or provide a list of excluded studies with reasons. The synthesis was narrative rather than meta‐analytical, and publication bias was not evaluated. Funding and conflicts of interest for primary studies were also not reported.

Haghnejad et al. [18] did not apply AMSTAR‐2, as the review focused on prediction model appraisal rather than biomarker meta‐analysis, but its methodological features can still be benchmarked against AMSTAR‐2 domains. The review was prospectively registered in PROSPERO and followed PRISMA and TRIPOD‐SRMA guidelines, with duplicate screening and risk‐of‐bias assessment using PROBAST. A comprehensive MEDLINE search was conducted, supplemented by reference tracking, but no additional databases were searched. The synthesis was narrative owing to heterogeneity, with discrimination and calibration metrics qualitatively summarized rather than pooled, and publication bias was not evaluated. Excluded studies were listed by reason, but the review did not provide details on funding or conflicts of interest for included primary studies. Overall, while the methodological rigor was strong in terms of protocol registration, transparent reporting, and structured risk‐of‐bias assessment, the absence of meta‐analytical synthesis, limited database coverage, and incomplete reporting of primary study funding/COI temper confidence in the review’s comprehensiveness.

Across all four reviews, the most common methodological weaknesses were the lack of reporting of primary study funding and conflicts of interest (item 10 and item 16), which were absent in every case. The results highlight that, while recent reviews in this area generally employ rigorous search and synthesis strategies, greater attention to transparency in primary study characteristics and to prospective protocol registration (where absent) would further strengthen methodological quality.

### 3.3. Overlap Analysis of Included Systematic Reviews and Meta‐Analysis

**Figure 3:**
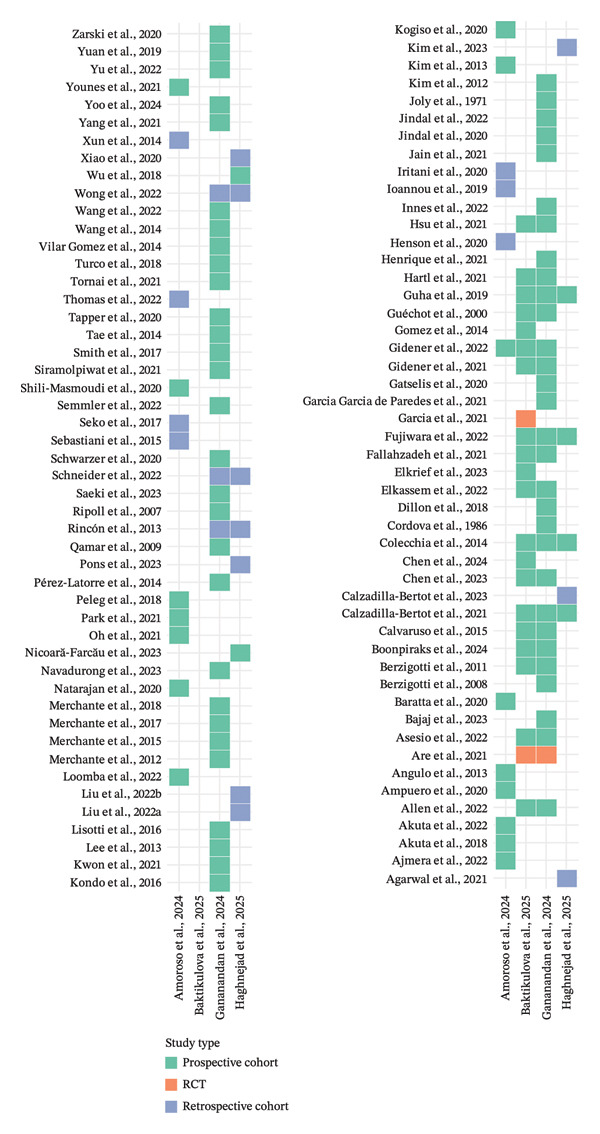
Heat map of primary study overlaps across included systematic reviews and meta‐analyses.

The degree of overlap among the primary studies included in the four systematic reviews was assessed to identify redundancy in the evidence base and to inform the interpretation of synthesized findings. Primary studies from each review were extracted, standardized to an “Author et al., Year” format, and cross‐referenced to identify common studies across reviews. Overall, overlap was moderate, with several primary studies appearing in more than one review, particularly those involving large, well‐characterized cohorts and widely used prognostic markers, such as LSM, MELD, and HVPG. A smaller subset of studies were present in all four reviews. The CCA, calculated using the formula proposed by Pieper et al. [19], indicated a moderate degree of overlap. This suggests that while the reviews drew on partly distinct primary literature—reflecting differences in inclusion criteria, etiology focus, and biomarker scope—there is sufficient duplication to warrant consideration when interpreting pooled results and making recommendations for future research. A detailed list of all primary studies, their study type, and the systematic review(s) in which they appeared is presented in Figure 3. This figure allows visual inspection of shared and unique studies across the four included reviews.

## 4. Discussion

This umbrella review synthesized evidence from four high‐quality systematic reviews—two with meta‐analyses and one narrative review—on prognostic biomarkers for predicting decompensation in patients with compensated cirrhosis of alcoholic and nonalcoholic etiologies. The findings highlight the substantial progress in identifying noninvasive and invasive markers capable of stratifying risk, while also revealing methodological and evidence gaps that limit direct translation into clinical practice.

Across the reviews, the most consistently predictive serum‐based markers included albumin, INR, bilirubin, platelet count, and inflammatory mediators, such as interleukin‐6 and keratin‐18. Imaging‐based parameters, particularly LSM via transient elastography and spleen size assessment, demonstrated robust prognostic value across etiologies. Hemodynamic measures, such as HVPG, retained their role as gold‐standard predictors, especially when combined with noninvasive measures. Composite scores—including MELD, ALBI, Child–Pugh, FIB‐4, APRI, and newer algorithms, such as MEFIB—offered improved predictive accuracy, particularly in multimarker models. Genetic markers, including PNPLA3 GG genotype, showed promise in NAFLD/NASH populations but require further validation before widespread adoption.

Etiology‐specific patterns emerged from the synthesis. In NAFLD–NASH populations, structural and functional markers tended to perform better, while in ALD, inflammatory markers and circulating extracellular vesicles appeared more predictive. These differences underscore the need for etiology‐tailored risk models that incorporate both shared and disease‐specific predictors.

Translation of novel biomarkers into routine cirrhosis care frequently fails due to multiple practical and methodological barriers. These include pre‐analytical and analytical variability, lack of standardized assays and outcome‐linked cut‐offs, limited interlaboratory reproducibility, cost and turnaround time constraints, and restricted access to specialized platforms, such as extracellular vesicle profiling or advanced omics technologies. In addition, biomarker performance is often population‐dependent, influenced by etiology mix, comorbidities, disease stage, and treatment exposures. Prognostic models may further lose performance when transported across clinical settings without recalibration and external validation, limiting their generalizability and clinical uptake.

Biomarker performance may also differ according to the type of first decompensation event, not solely by disease etiology. Ascites and variceal bleeding are primarily driven by portal hypertension and hemodynamic changes, whereas hepatic encephalopathy reflects systemic inflammation, neurotoxicity, sarcopenia, and altered ammonia metabolism. As a result, biomarkers related to portal pressure and liver stiffness may better predict ascites or variceal bleeding, while inflammatory or metabolic markers may be more relevant for encephalopathy. Because many included reviews reported composite outcomes of first decompensation, interpretation of biomarker performance for specific events should be approached cautiously, and future studies should report event‐specific prognostic performance to enhance clinical applicability.

The overlap analysis indicated a moderate CCA, suggesting partial redundancy among primary studies. Shared studies were often large, longitudinal cohorts or trials evaluating well‐established markers, which may bias pooled estimates toward frequently studied parameters. However, a significant proportion of studies were unique to each review, reflecting variability in inclusion criteria, population focus, and biomarker scope. This heterogeneity likely contributes to the observed variation in pooled effect sizes, particularly for imaging and inflammatory markers.

From a methodological perspective, the AMSTAR‐2 appraisal revealed strengths in comprehensive search strategies, clear PICO definitions, and appropriate synthesis methods in most reviews. However, common weaknesses included the lack of reporting on primary study funding sources and conflicts of interest, which may influence bias assessment. Only one review failed to preregister a protocol, but this limitation was sufficient to downgrade its confidence rating to low, according to AMSTAR‐2 guidance.

Clinically, these findings reinforce the concept that no single biomarker can reliably predict decompensation across all compensated cirrhosis populations. The strongest predictive performance was achieved when multidomain models—combining biochemical, imaging, and hemodynamic parameters—were applied. This supports current guideline trends toward integrated risk stratification, while also indicating that certain emerging biomarkers may enhance predictive models if validated in diverse populations. Beyond summarizing existing reviews, this umbrella review provides clinicians with an evidence strength–based hierarchy of prognostic biomarkers, clarifying which markers can be confidently used in routine risk stratification and which should be considered exploratory. By integrating certainty of evidence, heterogeneity, and methodological quality, this synthesis supports more cautious interpretation of emerging biomarkers and emphasizes the continued primacy of validated clinical and imaging‐based predictors.

It is important to distinguish between statistical association, discriminatory performance, and clinical usability when interpreting prognostic biomarkers. Many biomarkers included in this umbrella review—particularly inflammatory markers, extracellular vesicles, and genetic variants—demonstrated statistically significant associations with decompensation risk (expressed as HRs or ORs). However, association alone does not equate to predictive accuracy.

Discriminatory performance, assessed using AUROC or C‐statistics, was inconsistently reported and often derived from heterogeneous or methodologically limited studies. Furthermore, clinical usability depends on factors beyond statistical performance, including assay standardization, availability, validated cut‐offs, cost, and turnaround time. Consequently, several emerging biomarkers should be considered promising but exploratory, rather than robust predictors ready for routine clinical implementation.

While individual systematic reviews describe prognostic biomarkers within specific etiologies or methodological scopes, this umbrella review offers clinicians an integrated, evidence strength–based perspective across reviews. By comparing findings across heterogeneous reviews, we identify biomarkers whose prognostic associations remain stable across etiologies and study designs, notably routine laboratory markers (albumin, INR, bilirubin, and platelet count) and LSM.

Importantly, the application of an a priori evidence strength framework enables differentiation between biomarkers supported by consistent, moderate‐to‐high certainty evidence and those whose apparent prognostic value is limited by heterogeneity, low certainty, or methodological bias. This distinction is not explicit in individual reviews and is critical for clinical decision‐making.

The clinician‐facing ranking table (Table 3) translates this synthesis into a pragmatic format, highlighting which biomarkers are readily implementable in routine practice versus those that remain investigational. Finally, by synthesizing PROBAST assessments across reviews, this umbrella review clarifies common pitfalls in prognostic model development—such as limited external validation and inadequate calibration—thereby guiding cautious interpretation of complex prediction models in clinical settings.

### 4.1. Recommendations for Future Research

Future research should prioritize the external validation of promising biomarkers and prediction models in independent, multiethnic cohorts to ensure generalizability across diverse populations. Prospective, head‐to‐head comparisons of established and emerging markers are needed to determine their relative predictive performance across different etiologies of compensated cirrhosis. The development of dynamic risk models that incorporate longitudinal changes in biomarker values could enhance the accuracy of decompensation risk prediction over time. In addition, exploring biomarker panels that integrate inflammatory, metabolic, and genetic signals with standard clinical measures may yield more comprehensive and precise prognostic tools. Finally, improving transparency in reporting the funding sources and conflicts of interest for primary studies is essential to strengthen risk‐of‐bias assessments and enhance the credibility of the evidence base.

### 4.2. Common Methodological Flaws in Prognostic Model Development and Minimum Standards for Future Models

Although PROBAST was applied in the included reviews, critique of prognostic models has often remained descriptive rather than prescriptive. Across the reviewed prediction models for first decompensation, several recurring methodological weaknesses were identified. These included reliance on univariable screening for predictor selection, inadequate handling of missing data (often complete‐case analysis), small event‐per‐variable ratios leading to overfitting, inconsistent outcome definitions, and limited reporting of calibration performance.

Importantly, external validation was infrequently performed and, when conducted, was often limited to closely related populations rather than geographically or etiologically distinct cohorts. Calibration—an essential component of prognostic accuracy—was inconsistently reported, with few studies providing calibration plots, slopes, or intercepts. Moreover, very few models assessed clinical utility using decision‐curve analysis or net benefit metrics, limiting insight into whether improved discrimination translates into better clinical decision‐making.

Based on these findings, minimum methodological requirements for future prognostic models in compensated cirrhosis should include the following: (i) development and validation in adequately sized cohorts with sufficient events per predictor; (ii) transparent handling of missing data using appropriate imputation methods; (iii) comprehensive reporting of both discrimination and calibration; (iv) external validation in independent, geographically distinct populations; (v) model updating or recalibration when transported across settings; (vi) assessment of clinical utility using decision‐curve analysis; and (vii) transparent reporting in accordance with TRIPOD guidelines. Adoption of these standards is essential to improve model reliability and facilitate clinical implementation.

### 4.3. Limitations

The main limitation of this umbrella review is the reliance on existing systematic reviews, meaning that any biases or errors in the original reviews are inherently carried forward. Heterogeneity in study populations, definitions of decompensation, and biomarker cut‐offs across the included reviews limited direct comparability of pooled estimates. Furthermore, some potentially relevant biomarkers were underrepresented due to the time frames or search criteria of the included reviews.

## 5. Conclusion

This umbrella review synthesized evidence from four recent systematic reviews and meta‐analyses evaluating prognostic biomarkers for predicting decompensation in patients with compensated cirrhosis of alcoholic and nonalcoholic etiologies. The findings highlight that both established markers—such as albumin, INR, bilirubin, platelet count, and LSM—and emerging biomarkers, including interleukin‐6, keratin‐18, and extracellular vesicles, can provide valuable prognostic information. Imaging‐based, hemodynamic, and composite scoring systems consistently enhanced predictive accuracy, particularly when integrated into multimarker models.

While the evidence supports the clinical utility of multidomain risk stratification approaches, the methodological quality of existing reviews varied, and gaps remain in external validation, etiology‐specific modeling, and transparency in primary study reporting. Addressing these limitations through rigorous, prospective, and inclusive research will be critical for translating biomarker‐based prediction into routine clinical decision‐making, enabling earlier intervention and improved outcomes for patients with compensated cirrhosis.

## 6. Expert Opinion

This section presents the authors’ interpretation of the synthesized evidence and proposes a pragmatic approach for clinical application; it does not constitute a formal evidence grading beyond the umbrella framework described in the Methods. Across recent systematic reviews—including those with meta‐analyses—the most consistently informative signals for first decompensation in compensated cirrhosis remain foundational clinical and pathophysiologic measures: serum albumin, INR, bilirubin, platelet count, and noninvasive surrogates of portal hypertension, such as LSM. HVPG continues to represent the invasive reference standard, particularly when high‐impact decisions (e.g., transplant evaluation or consideration of preemptive TIPS) require precise hemodynamic assessment. Emerging inflammatory and cell injury markers, including interleukin‐6 and cytokeratin‐18, as well as circulating extracellular vesicles, appear promising as adjuncts but currently complement rather than replace established markers. Composite indices integrating multiple domains (e.g., MELD, ALBI, FIB‐4, APRI, MEFIB, and elastography‐augmented scores) generally outperform single biomarkers and better capture the multidimensional processes underlying decompensation.

From a practical standpoint, a tiered strategy is advisable. At baseline and during routine follow‐up (approximately every 3–6 months), readily available laboratory tests used to calculate MELD and ALBI, combined with LSM when accessible, provide a pragmatic foundation for risk stratification in most clinical settings. Escalation to HVPG should be considered when noninvasive assessments are discordant or when management decisions depend on accurate estimation of portal pressure. Etiology‐specific emphasis may further refine interpretation: In MASLD/NAFLD, structural and functional markers (LSM and composite indices) tend to discriminate risk more consistently, whereas in ALD, inflammatory signals (e.g., IL‐6, K18) may add incremental information where validated assays and thresholds are available. Importantly, biomarker results should be explicitly linked to predefined clinical actions—such as surveillance intensity for varices, initiation of nonselective beta‐blockers when indicated, optimization of diuretic therapy, or timing of referral for transplant evaluation—so that prognostic assessment meaningfully informs care.

Several considerations warrant caution. Novel biomarkers, particularly extracellular vesicles, lack standardized pre‐analytical workflows, assay harmonization, and outcome‐linked cut‐offs, limiting their generalizability. Many machine learning–based models report favorable discrimination in selected cohorts but provide limited calibration data and little external validation; without demonstrated clinical utility through decision‐curve or net benefit analyses, routine adoption remains premature. Moreover, single time‐point assessments may inadequately reflect the dynamic course of cirrhosis; longitudinal trends—such as declining albumin or rising INR or LSM—are likely more informative for near‐term risk than static thresholds. Methodological features of the evidence base, including moderate overlap of primary studies, heterogeneous definitions of first decompensation, variable cut‐offs, and inconsistent reporting of funding or conflicts of interest, further constrain transportability of results.

Looking forward, priorities include standardizing outcome definitions in line with Baveno VII, establishing clinically actionable cut‐offs for emerging biomarkers, and externally validating parsimonious models across diverse, multiethnic cohorts with transparent reporting of discrimination, calibration, and reclassification metrics. Incorporating repeated measures to enable dynamic risk updating and conducting head‐to‐head, etiology‐specific comparisons—while accounting for modifiable exposures, such as alcohol abstinence, weight loss, and pharmacologic interventions—will be essential. Ultimately, implementation considerations, including usability, turnaround time, and cost‐effectiveness, will determine real‐world impact, particularly in settings with limited access to elastography or HVPG.

In summary, the most reliable near‐term approach to predicting decompensation in compensated cirrhosis is an integrated, multidomain assessment anchored by routine laboratory measures and LSM, with HVPG reserved for pivotal decisions. Emerging inflammatory and vesicle‐based biomarkers are encouraging adjuncts but require further standardization and validation. Clinicians should preferentially use validated tools linked to actionable care pathways that influence surveillance, therapy, and transplant timing, thereby balancing prognostic accuracy with feasibility and equity.

## Author Contributions

Conceptualization, Amin Tamadon, Kristina Baktikulova, and Saulesh Kurmangaliyeva. Methodology, Amin Tamadon, Kristina Baktikulova, Saulesh Kurmangaliyeva, Ramazon Safarzoda Sharoffidin, and Kairat Kurmangaliyev. Investigation, Kristina Baktikulova, Saulesh Kurmangaliyeva, and Kairat Kurmangaliyev. Data curation, Saulesh Kurmangaliyeva, Kairat Kurmangaliyev, Ramazon Safarzoda Sharoffidin, and Kristina Baktikulova. Formal analysis, Kairat Kurmangaliyev and Kristina Baktikulova. Software, Kairat Kurmangaliyev. Validation, Kristina Baktikulova and Kairat Kurmangaliyev. Visualization, Kairat Kurmangaliyev. Writing–original draft, Kristina Baktikulova and Saulesh Kurmangaliyeva. Writing–review and editing, Kristina Baktikulova, Saulesh Kurmangaliyeva, Kairat Kurmangaliyev, Nadiar M. Mussin, Ramazon Safarzoda Sharoffidin, and Amin Tamadon. Supervision, Amin Tamadon. Project administration, Amin Tamadon. Resources, Nadiar M. Mussin and Amin Tamadon.

## Funding

This research was funded by the West Kazakhstan Marat Ospanov Medical University as part of the intra‐university project “Evaluation of the intensity of eryptosis processes and its role in the development of anemia and hemostasis disorders in patients with liver cirrhosis of HBV, HCV‐etiology.”

## Ethics Statement

This study is an umbrella review that synthesizes data from previously published studies. No primary data were collected from human participants by the authors. Therefore, ethical approval was not required for this research. However, all included studies were reviewed to ensure that they had obtained ethical approval from their respective institutional review boards and complied with international ethical standards. No ethical approval was required because this umbrella review used only published aggregate data.

## Consent

The authors have nothing to report.

## Conflicts of Interest

The authors declare no conflicts of interest.

## Supporting Information

Table S1. Overview of included systematic reviews and meta‐analyses on prognostic biomarkers/prediction models for predicting decompensation in alcoholic and non‐alcoholic patients with compensated cirrhosis.

Table S2. Methodological and analytical details of included systematic reviews and meta‐analyses on prognostic biomarkers/prediction models for predicting decompensation in alcoholic and non‐alcoholic patients with compensated cirrhosis.

## Supporting information


**Supporting Information** Additional supporting information can be found online in the Supporting Information section.

## Data Availability

The original contributions presented in the study are included in the article material; further inquiries can be directed to the corresponding author.
