# Supplementary material for: Prognostic Biomarkers for Predicting Decompensation in Alcoholic and Nonalcoholic Patients With Compensated Cirrhosis: An Umbrella Review
Source: Biochem Res Int. 2026 Feb 2;2026:9919068. doi: 10.1155/bri/9919068 (PMC12862496; doi:10.1155/bri/9919068)
Supplement: Supplementary file 1 — Supporting Information Additional supporting information can be found online in the Supporting Information section. [file BRI-2026-9919068-s001.docx]

Table S1. Overview of included systematic reviews and meta-analyses on prognostic biomarkers/prediction models for predicting decompensation in alcoholic and non-alcoholic patients with compensated cirrhosis

| Author, Year, (Reference) | Journal | Review Type | Population & Setting | Etiology Focus | Decompensation Outcomes | No. of Primary Studies | Total Participants | Biomarker Category | Specific Biomarkers / Predictors Assessed | Measurement Method | Key Conclusions |
| --- | --- | --- | --- | --- | --- | --- | --- | --- | --- | --- | --- |
| Amoroso et al., 2024 (1) | Journal of Molecular Medicine | SR | Adults with NASH cirrhosis or NAFLD F3–F4, observational longitudinal studies | NAFLD–NASH | Liver-related events (LREs: ascites, HE, VB, HCC), CVEs, mortality | 23 | Not reported | Serum-based, Imaging-based, Composite, Genetic | NFS, FIB-4, APRI, BARD, ELF, HFS, ALT/AST, AFP, platelet count, NLR, absolute lymphocyte count, sLOXL2, miR-122, LSM, MEFIB, PNPLA3 GG genotype | Serum assays, imaging (TE, MRE), composite scores, genetic testing | Multiple non-invasive biomarkers prognostic in NASH cirrhosis; ELF, LSM, APRI, MEFIB useful for treatment monitoring; ethnicity-specific thresholds needed |
| Baktikulova et al., 2025 (2) | Frontiers in Medicine | SR+MA | Adults with compensated cirrhosis (Baveno VII), observational cohorts & RCT control arms | ALD, NAFLD–NASH | Ascites, hepatic encephalopathy (HE), variceal bleeding (VB) | 66 | 37,063 | Serum-based, Imaging-based, Inflammatory, Hemodynamic | PVD, spleen size, bilirubin, albumin, MELD, INR, platelets, LSM, HVPG, IL-6, keratin-18, EVs | Imaging (US, elastography, CT/MRI), serum assays, HVPG | Etiology-specific markers improve prediction; supports integrated risk models |
| Gananandan et al., 2024 (3) | BMJ Open Gastroenterology | SR+MA | Adults with compensated cirrhosis (Baveno VII), observational cohorts, case-control, RCT control arms | Mixed | Ascites, HE, VB | 63 | 31,438 | Serum-based, Imaging-based, Hemodynamic, Composite, Physiological | Platelets, MELD, albumin, INR, bilirubin, AST/ALT ratio, CP, ALBI, ALBI-FIB4, CHESS-ALARM, EPOD, HVPG, liver stiffness, spleen size, LS ratio, LSN, BMI, LFI | Serum assays, elastography, CT/MRI, Doppler US, HVPG | INR, albumin most predictive; imaging/HVPG reliable; suggests multi-biomarker models |
| Haghnejad et al., 2025 (4) | Hepatology | Systematic Review | Adults with compensated advanced chronic liver disease (cACLD) or compensated cirrhosis; retrospective and prospective cohorts | Mixed etiologies (ArLD, MASLD, HBV, HCV, others); no model specifically for ArLD | First liver decompensation (ascites, HE, variceal bleeding, SBP, liver-related death, sometimes combined with HCC or transplantation) | 16 | 92 to 53,038 (depending on study; not pooled) | Serum-based, Imaging-based, Composite, Machine learning, Genetic/Metabolomic | Albumin, platelets, age, bilirubin, INR, liver stiffness (VCTE, MRE), presence of varices, MELD, FIB-4, ALBI, HVPG, endoscopy findings, metabolomics (methionine, ceramide, secretome signatures) | Clinical/lab assays, imaging (TE, MRE, spleen stiffness), endoscopy, metabolomics, machine learning algorithms | Most models showed moderate discrimination (AUC 0.75–0.92). Highest performing included elastography-based (MRE, ABC algorithm) and composite models (ALBI-FIB4, ANTICIPATE-NASH-LRE). However, 13/16 studies rated high risk of bias (PROBAST). No model ready for clinical implementation; heterogeneity in outcome definitions, reliance on non-routine predictors, limited external validation. Standardized definitions (Baveno VII), routinely available predictors, and etiology-specific validation are needed before clinical adoption. |

Abbreviations: AFP, alpha-fetoprotein; ALBI, albumin–bilirubin score; ALBI-FIB4, albumin–bilirubin plus fibrosis-4 combined score; ALD, alcohol-related liver disease; ALT, alanine aminotransferase; APRI, AST to platelet ratio index; AST, aspartate aminotransferase; BARD, BMI–AST/ALT ratio–diabetes score; BMI, body mass index; CHESS-ALARM, Cirrhosis Health Economic Scoring System–ascites, liver function, age, renal function, MELD; CP, Child–Pugh score; CT, computed tomography; CVEs, cardiovascular events; ELF, Enhanced Liver Fibrosis score; EPOD, Enhanced Prediction of Decompensation; EVs, extracellular vesicles; FIB-4, fibrosis-4 index; HCC, hepatocellular carcinoma; HFS, hepatic fibrosis score; HE, hepatic encephalopathy; HVPG, hepatic venous pressure gradient; IL-6, interleukin-6; INR, international normalized ratio; LFI, liver frailty index; LREs, liver-related events; LS, liver stiffness; LSM, liver stiffness measurement; LS ratio, liver stiffness to spleen stiffness ratio; LSN, liver–spleen ratio in nodularity index; MELD, Model for End-Stage Liver Disease; MEFIB, magnetic resonance elastography plus fibrosis-4 index; miR-122, microRNA-122; MRI, magnetic resonance imaging; NAFLD, non-alcoholic fatty liver disease; NASH, non-alcoholic steatohepatitis; NFS, NAFLD fibrosis score; NLR, neutrophil-to-lymphocyte ratio; PVD, portal vein diameter; PNPLA3, patatin-like phospholipase domain-containing protein 3; RCT, randomized controlled trial; sLOXL2, soluble lysyl oxidase-like 2; SR, systematic review; SR+MA, systematic review plus meta-analysis; TE, transient elastography; US, ultrasound; VB, variceal bleeding.

Table S2. Methodological and analytical details of included systematic reviews and meta-analyses on prognostic biomarkers/prediction models for predicting decompensation in alcoholic and non-alcoholic patients with compensated cirrhosis

| Author, Year, (Reference) | Effect Size Metric | Pooled Estimate (95% CI) | Statistical Model | Heterogeneity (I² / τ²) | Publication Bias | Subgroup / Meta-regression Findings | Sensitivity Analysis Findings | Review Risk of Bias (AMSTAR 2) | Primary-Study Risk of Bias Tool & Summary | Certainty of Evidence | Search Timeframe | Databases Searched | Funding & COI (of Review) |
| --- | --- | --- | --- | --- | --- | --- | --- | --- | --- | --- | --- | --- | --- |
| Amoroso et al., 2024 (1) | HR, OR, AUROC | NFS >0.676 HR up to 34.2; FIB-4 >2.67 HR up to 14.6; APRI >1.5 HR up to 20.9; ELF ≥11.27 HR 2.11; LSM ≥30.7 kPa HR 10.52; MEFIB positive HR >17; PNPLA3 GG HR 6.36 for HCC | Narrative synthesis | Not assessed | Not assessed | NFS/FIB-4 thresholds vary by ethnicity; ELF, APRI, LSM, MEFIB high potential; PNPLA3 GG genetic risk | Not assessed | High | PRISMA-based appraisal – majority moderate RoB | Moderate–High (GRADE) | Inception–Jan 2023 | OVID | Funded by Boehringer Ingelheim; authors employed by sponsor |
| Baktikulova et al., 2025 (2) | HR | PVD 7.39, spleen size 5.79, bilirubin 4.27, EVs 5.09, MELD (ALD) 1.87, MELD (NA) 1.13, K18 1.77, IL-6 1.31, LSM (NA) 1.26, HVPG 1.13 | Random-effects | Most biomarkers I² > 50%, HVPG 0% | Low (Egger’s test) | Structural/functional markers stronger in NAFLD–NASH; inflammatory (EVs, K18) stronger in ALD; IL-6 predictive in both | Robust under leave-one-out | High | QUIPS – majority low RoB | Moderate–High (GRADE) | Inception–Apr 2025 | PubMed, Scopus, Web of Science | Funded by West Kazakhstan Marat Ospanov Medical University; no COI |
| Gananandan et al., 2024 (3) | HR (log-transformed) | INR 0.76, albumin −0.35; HVPG ≥12–16 mmHg; LSM ≥13–40 kPa | Random-effects | High (I² up to 96.3%) | Egger’s p=0.58 | INR strongest, followed by albumin; imaging (spleen size, LSN) and HVPG robust; LSM predictive; novel scores promising | Not assessed | High | QUIPS – 34% low, 41% moderate, 25% high RoB | Moderate (GRADE) | Inception–Feb 2024 | PubMed, EMBASE | No specific funding; no COI |
| Haghnejad et al., 2025 (4) | Model discrimination (C-statistic / AUROC), calibration (plots, slopes, Brier score) | AUC values ranged 0.75–0.92 across studies (e.g., MRE-based 0.91 for 3y; ABC model 0.85–0.90) | Narrative synthesis; no meta-analysis | Not assessed | Not assessed | Subgroups: MASLD-only vs viral vs mixed cohorts; outcome definitions varied (ascites, VB, HE, SBP, HCC, death) | Not assessed | Not assessed | PROBAST used; 13/16 studies high overall ROB; common issues: univariable screening, missing data, lack of external validation | Low certainty (due to high ROB, heterogeneity, outcome inconsistencies) | Inception – Dec 2023 | MEDLINE via Ovid; handsearch of references | Funding: Association Française pour l'Étude du Foie, NIHR; COI: multiple authors reported consultancy/speaker fees from pharma (Gilead, AbbVie, Orphalan, Norgine, Bayer, Roche, Novo Nordisk) |

Abbreviations: ALD, alcohol-related liver disease; APRI, AST to platelet ratio index; AUROC, area under the receiver operating characteristic curve; COI, conflict of interest; ELF, Enhanced Liver Fibrosis score; EMBASE, Excerpta Medica database; EVs, extracellular vesicles; FIB-4, fibrosis-4 index; GRADE, Grading of Recommendations, Assessment, Development and Evaluations; HCC, hepatocellular carcinoma; HR, hazard ratio; HVPG, hepatic venous pressure gradient; I², Higgins and Thompson’s I-squared statistic; IL-6, interleukin-6; K18, keratin-18; kPa, kilopascal; LSF, liver stiffness measurement; LSN, liver–spleen ratio in nodularity index; MELD, Model for End-Stage Liver Disease; MEFIB, magnetic resonance elastography plus fibrosis-4 index; N/A, not applicable; NA, non-alcoholic; NAFLD, non-alcoholic fatty liver disease; NFS, NAFLD fibrosis score; OR, odds ratio; PNPLA3, patatin-like phospholipase domain-containing protein 3; PRISMA, Preferred Reporting Items for Systematic Reviews and Meta-Analyses; PVD, portal vein diameter; QUIPS, Quality in Prognosis Studies tool; RoB, risk of bias; τ², tau-squared statistic; OVID, Ovid database platform.

**References**

1. Amoroso M, Augustin S, Moosmang S, Gashaw I. Non-invasive biomarkers prognostic of decompensation events in NASH cirrhosis: a systematic literature review. J Mol Med (Berl). 2024;102(7):841-58. doi:10.1007/s00109-024-02448-2

2. Baktikulova K, Kurmangaliyeva S, Kurmangaliyev K, Tissin K, Mussin NM, Tamadon A. Prognostic biomarkers for predicting decompensation in alcoholic and non-alcoholic patients with compensated cirrhosis: A systematic review and meta-analysis. Frontiers in Medicine. 2025;12:1650124. doi:10.3389/fmed.2025.1650124

3. Gananandan K, Singh R, Mehta G. Systematic review and meta-analysis of biomarkers predicting decompensation in patients with compensated cirrhosis. BMJ Open Gastroenterol. 2024;11(1):e001430. doi:10.1136/bmjgast-2024-001430

4. Haghnejad V, Burke L, El Ouahabi S, Parker R, Rowe IA. Prediction models for liver decompensation in compensated advanced chronic liver disease: A systematic review. Hepatology. 2025. doi:10.1097/HEP.0000000000001359
